# Estimating cardiorespiratory fitness in older adults using the international physical activity questionnaire

**DOI:** 10.3389/fspor.2024.1368262

**Published:** 2024-06-24

**Authors:** Kelsey R. Sewell, Jeremiah J. Peiffer, Shaun J. Markovic, Belinda M. Brown

**Affiliations:** ^1^ Centre for Healthy Ageing, Health Futures Institute, Murdoch University, Murdoch, WA, Australia; ^2^ Australian Alzheimer’s Research Foundation, Sarich Neuroscience Research Institute, Nedlands, WA, Australia; ^3^ School of Medical and Health Sciences, Edith Cowan University, Joondalup, WA, Australia

**Keywords:** aged, cardiorespiratory fitness, fitness testing, non-exercise, estimate

## Abstract

**Introduction:**

Non-exercise estimates of cardiorespiratory fitness hold great utility for epidemiological research and clinical practice. Older adults may yield the greatest benefit from fitness estimates due to limited capacity to undergo strenuous maximal exercise testing, however, few of the previously developed non-exercise equations are suitable for use in older adults. Thus, the current study developed a non-exercise equation for estimating cardiorespiratory fitness in older adults derived from the widely used International Physical Activity Questionnaire (IPAQ).

**Methods:**

This study was a secondary analysis of baseline data from a randomized controlled trial. Participants were community-dwelling, cognitively unimpaired older adults aged 60–80 years (*n* = 92). They completed the IPAQ and underwent maximal exercise testing on a cycle ergometer. Stepwise linear regression was used to determine the equation in a randomly selected, sex-balanced, derivation subset of participants (*n* = 60), and subsequently validated using a second subset of participants (*n* = 32).

**Results:**

The final equation included age, sex, body mass index and leisure time activity from the IPAQ and explained 61% and 55% of the variance in the derivation and validation groups, respectively (standard error of estimates = 3.9, 4.0). Seventy-seven and 81% of the sample fell within ±1SD (5.96 and 6.28 ml·kg−1·min−1) of measured VO_2peak_ for the derivation and validation subgroups. The current equation showed better performance compared to equations from Wier et al. (2006), Jackson et al. (1990), and Schembre & Riebe (2011), although it is acknowledged previous equations were developed for different populations.

**Conclusions:**

Using non-exercise, easily accessible measures can yield acceptable estimates of cardiorespiratory fitness in older adults, which should be further validated in other samples and examined in relation to public health outcomes.

## 1 Introduction

Cardiorespiratory fitness (CRF) is associated with a lower risk for diabetes, cardiovascular disease, and all-cause mortality (1–3). Reflecting the overall health of the cardiovascular system, CRF is associated with health outcomes particularly important for older adults: better cognition, physical functioning, and lower falls risk (4, 5). The current “gold-standard” assessment of CRF is a graded exercise test with measurement of oxygen uptake (VO_2max_) and requires specialized equipment and trained personnel. Other CRF assessments include submaximal exercise testing and predictive exercise estimates such as the six-minute walk tests. However, these methodologies may be impractical for use in large epidemiological studies and, in some instances, primary care settings. Non-exercise estimates of CRF (eCRF) have emerged as an attractive alternative to maximal exercise tests and are currently recommended for use in primary care (6). Higher eCRF is associated with lower all-cause and cardiovascular disease mortality, and improved brain structure and function (7, 8). However, the accuracy of eCRF may depend on the population subgroup in which the equations are derived and utilized (9). A majority of current equations have been validated in cohorts with a mean age of 40–50 years and are less accurate when implemented in older adults (9, 10). This is problematic since older adults are the most at-risk population for health outcomes commonly associated with low CRF, and thus may benefit most from routine assessment of fitness using a non-exercise estimate.

There have been over 60 non-exercise fitness estimates developed to date, however few of these equations have been derived specifically for use in older adults (11). Limited generalizability of eCRF equations has been cited as a limitation in the literature (9), however, it is feasible, and important, to utilize population-specific equations when estimating eCRF, especially for different life stages, since fitness tends to decline in ageing (12). Two previous studies have developed older adult specific equations; however, one was in male participants only (13), and a recently published equation included data from a submaximal exercise test (i.e., the 6-min walk test) (14). Notably, the latter equation explained a high percentage (74%–87%) of the variance in measured cardiorespiratory fitness, yet these type of field tests are not always applicable, particularly for older adults in population-based studies. Further, maximal or submaximal measures of fitness often vary by protocol and exercise type, making direct comparisons difficult (e.g., 6-min walk test vs. modified Bruce treadmill test vs. Chester step test); thus, an equation which can accurately predict VO_2max_ and is comparable across studies in older adults is warranted.

Existing non-exercise CRF estimates use a combination of demographic characteristics such as age and sex, combined with anthropometric measures such as body mass index (BMI), resting heart rate, or body fat %. Some equations also include a measure of self-reported physical activity, the most commonly used is the NASA/Johnson Space Center (JSC) physical activity scale (PA-R) (15, 16), or a derivative of this scale (17). The widespread use of the PA-R in already established eCRF equations is challenging because outcomes from other self-report measures are not directly comparable to the PA-R. Given that physical activity is a determinant of CRF (18), correct estimation of this variable is important for an accurate eCRF equation. The International Physical Activity Questionnaire (IPAQ) is a valid, reliable, and widely-used alternative to the PA-R (19). Outcomes from the IPAQ, specifically vigorous activity, are correlated with objective physical activity (20), and the IPAQ is particularly useful in population-based samples, in which eCRF is also commonly implemented. To the best of our knowledge, there has only been one previous study (21) which developed an eCRF equation based on the IPAQ, and this was in college students. Because equation accuracy may depend on population subgroup (9), development of an equation specifically with the IPAQ in older adults is warranted.

Graded maximal exercise tests conducted on either a treadmill or cycle ergometer are used to validate eCRF equations. Majority of previous studies have validated their equations using a treadmill, and these protocols may produce 5%–10% higher VO_2max_ values compared to cycling (22). These higher VO_2max_ values may be due to the increased muscle mass engaged in running compared to cycling, or because of localized muscle fatigue from unfamiliar exercise resulting in premature test completion before reaching VO_2max_ (23). However, using cycling protocols is advantageous for pragmatic reasons, (ease of other concurrent measurements e.g., blood pressure, lower cost, and portability), and has particular utility for older adults since cycling can be used in individuals with orthopedic limitations, and is safer when considering falls risk (24). Therefore, an equation derived and validated specifically using a cycling protocol is necessary (9, 10).

The aim of the current study was to develop a non-exercise estimate of CRF, based on a cycling exercise test, for older adults using the IPAQ. Because of potential utility in population-based studies, we aimed to develop an equation using variables which can be fully self-reported. Performance of our equation was compared with previously high performing, cross-validated, equations. Specifically, we selected comparisons to Jackson et al. (15) and Wier et al. (16) equations based on their performance in older adults (10), and the Schembre & Riebe (21) equation because it was derived specifically for use with the IPAQ. We hypothesized that our estimates would predict directly measured CRF, and that they would perform better than previous equations due to our sample specificity and methodology differences (cycling protocol in the current study vs. treadmill protocols in previous studies). Validation of our equation would allow its use with similar populations in large, epidemiological studies.

## 2 Methods

### 2.1 Participants

Participants were drawn from the Intense Physical Activity and Cognition (IPAC) study (25). The IPAC study was a randomised clinical trial, however only baseline data are used for the current analyses (regardless of intervention allocation). The IPAC inclusion criteria have been detailed previously (25); briefly, participants were aged 60–80 years, community-dwelling, and cognitively unimpaired. The total sample for the IPAC study is *n* = 99, however 7 participants had incomplete physical activity questionnaire data and were excluded from the current analyses (resulting *n *= 92).

Written informed consent was obtained prior to participation and ethical approval was granted by the Human Research Ethics Committees at Murdoch University and Edith Cowan University. The IPAC study is registered with the Australian New Zealand Clinical Trials Registry (ACTRN12617000643370).

### 2.2 Physical activity questionnaire

Habitual physical activity levels were assessed using the IPAQ which is a self-report measure of physical activity behaviours over the last seven days (19). The questionnaire measures physical activity in four domains: work, housework, leisure time, and transportation activities. The IPAQ was scored following the standard recommendations (19). Namely, a Metabolic Equivalent of Task score (MET) is allocated to each question response based on activity intensity (e.g., walking = 3.3, moderate activities = 4, vigorous activities = 8), and a seven-day activity score is calculated (MET minutes/week). Domain-specific scores are also calculated by isolating METs for each domain (i.e., a separate MET score for physical activity during leisure time, work, housework and transportation). Using the standard instructions, the IPAQ was also scored to yield three physical activity classifications (low, moderate and high activity levels) using the same parameters previously described (26).

### 2.3 Non-exercise prediction equations

We developed our own non-exercise cardiorespiratory fitness equation using the IPAC cohort. Performance of this equation was compared to three previously validated non-exercise cardiorespiratory fitness equations. Of note, the Wier (16) (BMI) and Jackson (15) (BMI) equations have been developed for use with the NASA/Johnson Space Center (JSC) physical activity scale. We substituted this variable for physical activity classifications based on the IPAQ (scored as above). The Schembre & Riebe (21) equation has been developed specifically for use with the IPAQ (using the total METs from vigorous activity score). However, this equation was developed using college students and the best performing model reported in that study did not include age. Given our sample is older adults and age is a significant predictor of lower fitness in this population, we utilised the second equation reported in Schembre & Riebe (21) study with a negative weighting for age. Notably, all previous equations were derived and validated using treadmill exercise protocols. Each equation is detailed in Supplementary Table S1.

### 2.4 Direct fitness assessment

Direct measurement of cardiorespiratory fitness was conducted by a cycling-based graded exercise test was used to quantify peak aerobic capacity (VO_2peak_). VO_2peak_ was used rather than VO_2max_ (commonly used in other validation studies) because the occurrence of a plateau in VO_2_ which defines the VO_2max_ is observed in <50% of the general population, and even less so in older adults (27). The test used 2-minute stage durations with consistent increases in work rate at each stage, based on participants body mass, until participants reached volitional fatigue. During the test, heart rate and ventilatory gases were continuously recorded and averaged into 15-second intervals by a metabolic cart (TrueOne 2400, Parvomedics). VO_2peak_ was defined as the greatest consecutive 15-second mean values during the final two minutes of the test. Additional criteria for the assessment of VO_2peak_ involve participants reaching a maximal heart rate greater than 85% of their age predicted maximum [i.e., (220 − 2 age) × 0.85] and a respiratory exchange ratio (VCO_2_/VO_2_) greater than 1.15. For a full description of the fitness assessment see Brown et al. (25).

### 2.5 Statistical analyses

All analyses were conducted using R statistical computing packages version 4.2.1 (28). Descriptive statistics were calculated to compare demographic variables between IPAC subgroups (total *n *= 92; derivation *n *= 60, validation *n *= 32). For continuous variables, *t*-tests were conducted to determine differences across study groups, whilst chi-square tests were conducted for categorical variables.

To derive our non-exercise cardiorespiratory fitness equation, the sample was randomly split into derivation (*n* = 60) and validation (*n *= 32) subgroups. These groups were gender-balanced and consideration was given to the distribution of VO_2peak_ in each group: we categorised VO_2peak_ into quantiles in the full sample, then ensured relatively even distribution across subsamples (*n* in derivation, Q1: 12, Q2: 16, Q3: 18, Q4: 14; *n* in validation, Q1: 6, Q2: 9, Q3: 8, Q4: 9). Variables of interest were selected if they are valid predictors of cardiorespiratory fitness in older adults, and have performed well in previously validated equations, and included age, sex, body mass index, and self-reported physical activity. In line with our primary aim, we wanted to determine which IPAQ outcome was most suitable for equation use in older adults, so we considered: total IPAQ METs, moderate IPAQ METs, vigorous IPAQ METs, total walking minutes, leisure time IPAQ METs, and the physical activity category derived from the IPAQ. Pearson's *r* was used to determine significant correlations between the respective variable of interest and VO_2peak_ in the derivation group, and only variables which were significantly correlated with VO_2peak_ were considered for the equation.

Firstly, we used stepwise linear regression to determine our estimation equation, including the significantly correlated variables of interest (from above) as predictors and VO_2peak_ as the outcome. We then used this equation to estimate cardiorespiratory fitness in both the derivation and validation groups. To examine equation performance, separate linear regressions were run for each equation (Jackson (15), Schembre & Riebe (21), Wier (16), or the current study), using the respective equation as the predictor and VO_2peak_ as the outcome. We examined the adjusted *R*^2^ and standard error of the estimate (SEE) from the regression analyses as an indication of equation performance. Pearson's *r* was calculated for each equation to determine whether estimated and measured fitness were correlated, and *t*-tests were conducted to examine whether estimated and measured fitness significantly differed for each equation. These analyses were supplemented with visual inspection of Bland-Altman plots which indicate any patterns of error between estimated fitness and measured fitness. The mean difference between estimated and observed cardiorespiratory fitness and 95% confidence intervals were calculated for these plots.

## 3 Results

Participant demographics for the derivation and validation subgroups are presented in Table 1. The sample had a mean age of 68.9 ± 5.1 and were 53% female; there were no significant differences in demographics between derivation and validation subgroups.

**Table 1:** Descriptive statistics of the derivation and validation subsample of the IPAC cohort.

	Derivation subsample (*n* = 60)	Validation subsample (*n* = 32)	Test statistic
Age, years	68.7 (5.4)	69.8 (5.2)	*t *= −1.01
Sex, % female (*n*)	50 (30)	46.9 (15)	*χ^2^* = 0
*APOE ε*4 allele carriers, % (*n*)	18.3 (11)	28.1 (9)	*χ^2^* = 0.67
Years of education	13.9 (2.3)	14.3 (2.2)	*t *= −0.91
Global cognition	26.8 (2.1)	26.6 (2.2)	*t *= 0.32
Alcohol, units per week	6.4 (6.1)	4.8 (5.6)	*t *= 1.31
Depression score	1.6 (1.9)	2.2 (3.2)	*t *= −1.01
Body mass index (kg/m^2^)	25.8 (3.4)	25.8 (4.2)	*t *= 0.03
Total physical activity METs from IPAQ	4,219.1 (2,428.4)	4,085.1 (2,643.8)	*t *= 0.24
Leisure Time METs from IPAQ	1,237.8 (1,346.8)	1,117.6 (1,463.8)	*t *= 0.39
Baseline VO_2_peak (ml/kg/min)	23.5 (6.3)	24.1 (6.0)	*t *= −0.46
Estimated cardiorespiratory fitness^a^	23.5 (4.9)	22.9 (5.5)	*t *= 0.51

Unless otherwise described, data are presented as mean (standard deviation). Differences between groups were calculated using independent samples *t*-tests for continuous variables and chi-square for categorial variables. There were no significant differences between groups. Global cognition was assessed using the Montreal Cognitive Assessment. IPAC, intense physical activity and cognition; *APOE*, apolipoprotein E gene; VO_2peak_, volume of oxygen uptake during peak exercise; IPAQ, international physical activity questionnaire; METs, metabolic equivalent of task.

^a^ Derived from the current study's equation.

### 3.1 Estimation of a non-exercise fitness equation

Within the derivation subset, only vigorous activity METs and leisure time METs from the IPAQ were correlated with VO_2peak_ (*r* = .28, *p *= .039; *r* = .36, *p *= .005, respectively; Supplementary Table S2). Age (*r* = −.39), sex (*r* = .43), and body mass index (*r* = −.37), all showed significant correlations with VO_2peak_ (*p* < .01; Supplementary Table S2). Stepwise regression conducted with the derivation subset showed that age, sex, body mass index and leisure time physical activity (IPAQ METs) were the most suitable variables for VO_2peak_ estimation, all with significant individual contribution (Supplementary Table S3). The total model explained 59% of the variance in VO_2peak_ (SEE = 4.0), and the resultant equation was: 67.584 − (0.463 × Age) + (6.783 × Sex, 0 = Female, 1 = Male) + (0.075 × √IPAQ Leisure METs) − (0.692 × body mass index) (Supplementary Table S3).

### 3.2 Comparison of non-exercise cardiorespiratory fitness equations

Cardiorespiratory fitness estimates derived from Wier (16), Jackson (15), Schembre & Riebe (21) and the derviation and validation samples in the current study were all correlated with directly measured VO_2peak_ (*p* < .001; Table 2). The Schembre & Riebe (21) estimate explained 46% of the variance in measured VO_2peak_, the Jackson (15) estimate explained 42% of variance, while the Wier (16) equation explained 45% of the variance (Table 2). For the current study, our equation explained 61% of the variance in the derivation group, and 55% in the validation group. Estimates derived from the Schembre & Riebe (21) and Jackson (15) equations significantly differed from measured VO_2peak_ (*p *< .001, *p *= .008, respectively; Table 2), however estimates derived from the Wier (16) equation (*p *= .09), and the derivation and validation groups from the current study did not (*p *= .99, *p *= .41, respectively; Table 2).

**Table 2:** Comparison of non-exercise cardiorespiratory fitness equations for estimating VO_2_peak in the IPAC sample.

	*r*	Adjusted *R*^2^	Standard error of estimate	*t*-test value	Raw mean difference
Jackson et al. (1990)	.66	.42	4.65	*t* = 2.67*	2.4
Schembre & Riebe (2011)	.68	.46	4.51	*t* = 18.66**	14.4
Wier et al. (2006)	.67	.45	4.56	*t* = 1.68	1.4
Current study					
Derivation subgroup (*n* = 60)	.79	.61	3.92	*t* = −0.01	−0.01
Validation subgroup (*n* = 32)	.75	.55	4.01	*t* = 0.84	1.2

All estimated equations were significantly correlated with, and significant predictors of, VO_2peak_ at *p < *.001. *t*-tests compared measured VO_2peak_ with values derived from the respective equation. Mean difference calculated as VO_2peak_ − predicted VO_2peak_. *r*, correlation coefficient Pearson's *r*; *R*^2^, coefficient of determination from linear regression model; VO_2peak_, volume of oxygen uptake during peak exercise.

Bolded values indicate significance at *p* < .05.

* *p* < .05.

** *p* < .001.

**Figure 1:**
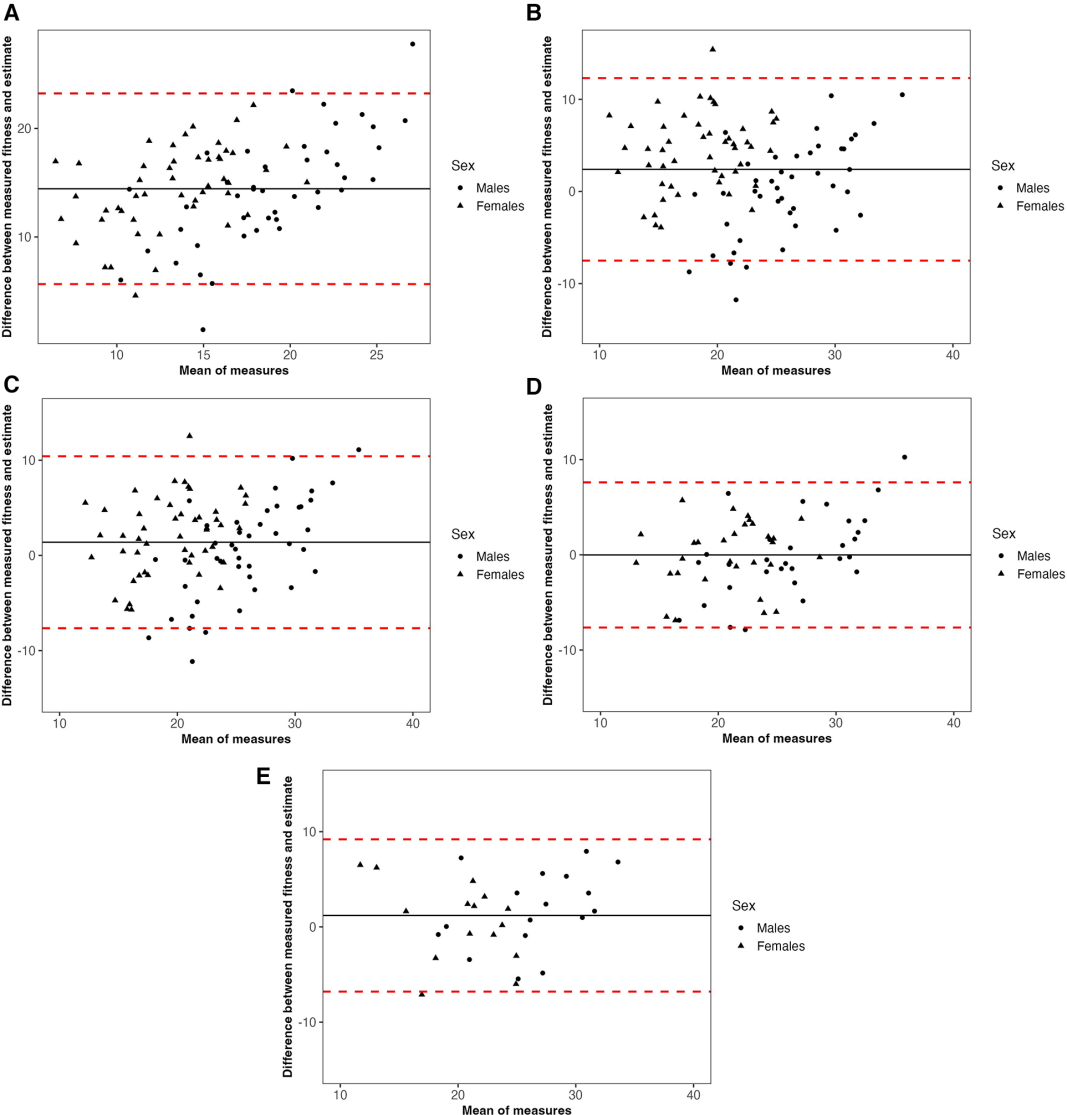
Bland-Altman plots for each equation between predicted and measured cardiorespiratory fitness. Solid black line represents the mean difference between estimated and observed cardiorespiratory fitness, dashed red lines indicate 95% confidence intervals for the mean difference. (**A**) Schembre & Riebe (21) (**B**) Jackson (15) (**C**) Wier (16) (**D**) Current study derivation group (**E**) current study validation group. (**A**) Mean difference between measured fitness and Schembre (solid black line) = 14.44. (**B**) Mean difference between measured fitness and Jackson estimate (solid black line) = 2.40. (**C**) Mean difference between measured fitness and Wier estimate (solid black line) = 1.38. (**D**) Mean difference between measured fitness and derivation group estimate (solid black line) = −0.01. (**E**) Mean difference between measured fitness and validation group estimate (solid black line) = 1.20.

The Bland-Altman plots (Figure 1) showed the Schembre & Riebe (21) estimate overestimated VO_2peak_ (majority of the datapoints fall above the zero-axis, high mean difference at 14.44). The Jackson (15) and Wier (16) estimates show similar performance, although from the Wier (16) estimate 75% of the sample fell within ±1SD (6.13 ml·kg^−1^·min^−1^) of measured VO_2peak_, while from the Jackson (15) equation only 62% of the sample fell within this range. For our derivation and validation subgroups, 77% and 81% of the sample fell within ±1SD (5.96 and 6.28 ml·kg^−1^·min^−1^) of measured VO_2peak_, respectively.

## 4 Discussion

The current study aimed to develop and validate a non-exercise fitness estimate in older adults using primarily self-reported data. Leisure time physical activity from the IPAQ, BMI, age, and sex were significant predictors of CRF, and our equation including these variables explained 61% and 55% of the variance in our derivation and validation subgroups, respectively. Our equation demonstrated acceptable performance for estimating CRF and will be valuable in epidemiological studies with similar older adult samples.

Validation results from our equation are comparable to generally accepted standards and previously reported results. In original validation studies, *R*^2^ values range from .43–.65 (see from 5.5–5.1; 17, 21), compared to .55–.61 from the current equation (SEE from 3.9–4.0). Wier (16) and Jackson (15) both do not report *R*^2^ values in their original papers but demonstrated similar correlation coefficients to our equation in the current study (*r* = .78 –.81; current equation *r* = .75 –.79). Within our validation group, only one participant (3% of the sample) fell outside of the 95% confidence interval for our estimate, suggesting reliable estimation not driven by outliers.

Submaximal exercise testing (e.g., Rockport 1-mile walk test) is often utilized as an alternative to maximal exercise testing for estimating VO_2peak_. Importantly, previous studies have found non-exercise eCRF to be as accurate for estimating VO_2peak_ as submaximal exercise testing but with lower participant burden (i.e., no exercise required) (15). The generally accepted error rate for submaximal exercise testing to estimate VO_2peak_ is between 10%–20% (16). In the current study, 78% of the individual estimates fell within 20% of the observed VO_2peak_, suggesting similar performance to submaximal testing from our eCRF, with lower participant burden. However, it should be acknowledged that similar to previous studies (21), our equation may be less accurate for those with either very low or high CRF, demonstrated by slight deviation in these areas in the Bland-Altman plots. Specifically, for less fit individuals, our equation tended to overestimate CRF compared to direct measurement, and for fitter individuals, tended to underestimate CRF (see Figure 1D). However, it is important to note that in less fit individuals, measured VO_2peak_ may be confounded by fatigue, unfamiliarity with testing protocols, or exercise type, resulting in these individuals not reaching their “true” VO_2peak_ via direct measurement. Thus, as acknowledged in previous reviews, population specifics i.e., fitness level and age group, should be considered when selecting an appropriate eCRF equation (11).

The equation developed in the current paper may be particularly useful in large, population based observational studies, many of which use the IPAQ to measure physical activity. It is widely acknowledged that individuals tend to overreport physical activity in self-reported measures (29). Importantly for older adults, this overreporting tends to be greater in those with worse cognition (30). One advantage of using the IPAQ over the PA-R is that subdomains of the IPAQ can be used to try and circumvent this issue, because examining a single domain provides limited opportunity for overreporting. Indeed, we observed the strongest correlation between leisure time physical activity and VO_2peak_ in the current study (Supplementary Table S2). This differs from previous research which found the strongest association between vigorous activity from the IPAQ and CRF, or categorizations (i.e., low, moderate and high activity) from the IPAQ and CRF (21). These studies have been conducted in younger or middle-aged adults, and because of lifestyle changes which often accompany retirement (i.e., greater leisure time), the leisure time variable may be more accurate for estimating CRF in older adults specifically (indeed, the current sample consisted of 79% retired individuals). Additionally, our results may differ from previous studies examining IPAQ categorizations because majority (62%) of our sample fell into the “high” physical activity category, resulting in a lack of adequate discrimination between activity levels.

In the current study, Wier (16), Jackson (15), and Schembre & Riebe (21) equations showed poorer performance for predicting measured VO_2peak_ than reported in the original validation studies. This is likely due to sample differences and use of a cycling protocol in the current study, as opposed to a treadmill protocol. Although our equation performed best within our sample, the Wier (16) equation also showed acceptable performance (Table 2). This is consistent with a previous study which validated the Wier (16) equation in older adults (10). However, in our case it is somewhat surprising since the Wier (16) equation requires self-reported physical activity categorization and there were small numbers of individuals within each of the three IPAQ categories in our study. Future studies using the PA-R in older adults may consider using the Wier (16) equation in this population, particularly if they do not have access to resting heart rate data which the Jurca (17) equation requires.

Unfortunately, because this study was a secondary analysis and we had limited data availability, we were restricted in the number of non-exercise equations we could compare [e.g., the Jurca (17) equation requires resting heart rate data]. Other equations by Jackson (31) and Wier (16) require body fat %, however, such equations show similar validity to using BMI (16), and because our aim was to develop a self-reported equation, we did not include this measure. Since the weighting for each factor is derived with all variables included in the model, it is not feasible to preserve equation integrity whilst omitting particular variables. However, limited time and resources is a challenge in both clinical and research settings, thus utilizing the minimum number of variables to limit participant and clinician burden, whilst maintaining equation accuracy, is beneficial.

A limitation of our study is the relatively homogenous sample. Since the current project was a secondary analysis from an exercise intervention study, volunteers were healthy, active, and highly motivated, which is reflected in the high percentage (62%) of individuals falling in the “high” IPAQ category. This may result in over-estimation of VO_2peak_ if our equation is applied to a more sedentary sample, and this limitation should be considered by future studies using the equation. Additionally, due to our limited sample size we were unable to derive and validate sex-specific equations, which may be more accurate than a single, general equation. However, we did test the sex-specific Wier (16) equations in a secondary analysis (data not shown), and they showed poorer performance than the original equation in our sample. Many population-based studies are interested in tracking changes in CRF over time, however, all non-exercise eCRF equations may be limited in their sensitivity to change since the factors which explain majority of the variance are stable over time (i.e., sex), and the variable most likely to change (self-reported physical activity), has the lowest weighting. Nevertheless, there are a lack of studies which have examined eCRF change over time (11), and this would be an interesting avenue for future research. We used a cycling protocol for evaluating VO_2max_ due to physical limitations in the older adult population, however, this may also increase differences between estimates from the current equation and those generated using a treadmill protocol. Finally, although our estimation explained an acceptable proportion of the variance in our sample (55%–61%), and can reliably estimate fitness in large samples, it may under- or over-estimate fitness at an individual level. For clinical practice, equations which include field-based measures such as Velázquez-Díaz (14) and can explain a greater proportion of variance (74%–87%), may be more suitable.

The current study derived and cross-validated a non-exercise estimate of cardiorespiratory fitness in older adults. Our final equation included sex, age, BMI and leisure time activity from the IPAQ, and explained 55% of the variance in our validation sample. The appeal of eCRF measures is clear: they are an efficient, cost-effective way to yield a general indication of fitness, and can be implemented in studies with thousands of participants where maximal exercise testing is not feasible. The IPAQ is a widely used and validated tool, is easy to administer, and using its subdomains may aid in reducing the overreporting of subjective physical activity. The current results contribute an eCRF equation specific to older adults using the IPAQ to measure physical activity, a population and measure in which the use of previous eCRF equations are not viable. Our equation will be utilized in future studies to examine associations between eCRF and important health outcomes, such as cognition, cardiovascular disease and falls risk in large, cohort-based research. This type of research will aid in widespread use of fitness measures and may help shape optimal physical activity recommendations for healthy aging in older adults.

## Data Availability

The data analyzed in this study is subject to the following licenses/restrictions: the datasets generated and/or analyzed during the current study are not publicly available due to additional secondary analyses currently being conducted but are available from the corresponding author on reasonable request. Requests to access these datasets should be directed to belinda.brown@murdoch.edu.au.
