# Supplementary material for: Estimating cardiorespiratory fitness in older adults using the international physical activity questionnaire
Source: Front Sports Act Living. 2024 Jun 24;6:1368262. doi: 10.3389/fspor.2024.1368262 (PMC11228262; doi:10.3389/fspor.2024.1368262)
Supplement: Supplementary file 1 [file Table1.docx]

# Supplementary Material

| Supplementary Table 1. Non-exercise cardiorespiratory fitness equations | |
| --- | --- |
| **Author** | **Equation** |
| Jackson et al. (1990) | 56.363 + [1.921 x IPAQ Category] – [0.381 x Age] – [0.754 x BMI] + [10.987 x Sex, 0=Female, 1=Male] |
| Schembre & Riebe (2011) | 55.955 – [6.553 x Sex, males = 1 and females = 2] – [0.334 x Age] – [0.504 x BMI] + [0.148 x (√IPAQ Vigorous Activity)^-2^] – [0.009 x (√IPAQ Moderate Activity)] – [0.090 x (√IPAQ Walking Minutes)] |
| Wier et al. (2006) | 57.402 – [0.372 x Age] + [8.596 x Sex, 0=Female, 1=Male] + [1.396 x IPAQ Category] – [0.683 x BMI] |
| Current study | 67.584 – [0.463 x Age] + [6.783 x Sex, 0=Female, 1=Male] + [0.075 x √IPAQ Leisure METs] – [0.692 x BMI] |
| *Note.* For Jackson and Wier equations, the NASA physical activity status scale was replaced with ratings from the IPAQ. Abbreviations: IPAQ, International Physical Activity Questionnaire; BMI, Body Mass Index; METs, Metabolic equivalent of task. | |

| Supplementary Table 2. Pearson Correlation of VO_2peak_ with independent variables in derivation group | | | | | | | | | | | | |
| --- | --- | --- | --- | --- | --- | --- | --- | --- | --- | --- | --- | --- |
|  | | *Age* | *BMI* | *Sex* | *IPAQ total walking minutes* | *IPAQ Vigorous Activity* | *IPAQ Leisure Time Activity* | *IPAQ Moderate Activity* | | *IPAQ Total Activity* | *IPAQ Category* |  |
| *Age* | - | |  |  |  |  |  | |  |  |  |  |
| *BMI* | .21 | | - |  |  |  |  | |  |  |  |  |
| *Sex* | .25 | | .14 | - |  |  |  | |  |  |  |  |
| *IPAQ total walking minutes* | -.04 | | -.04 | .09 | - |  |  | |  |  |  |  |
| *IPAQ Vigorous Activity* | -.12 | | .01 | .13 | -.07 | - |  | |  |  |  |  |
| *IPAQ Leisure Time Activity* | -.16 | | .10 | .19 | .45*** | .72*** | - | |  |  |  |  |
| *IPAQ Moderate Activity* | .26* | | .03 | .05 | .31* | .05 | .17 | | - |  |  |  |
| *IPAQ Total Activity* | .12 | | -.01 | .10 | .59*** | .41*** | .58*** | | .83*** | - |  |  |
| *IPAQ Category* | .17 | | -.02 | -.10 | .54*** | .12 | .31* | | .67*** | .73*** | - |  |
| *VO_2peak_* | -.39** | | -.37** | .43*** | .20 | .28* | .39** | | -.18 | .06 | -.03 |  |
| * *p* < .05; ** *p* < .01; *** *p* < .001. Abbreviations: BMI, body mass index; VO_2_peak, volume of oxygen uptake during peak exercise; IPAQ, International Physical Activity Questionnaire | | | | | | | | | | | | |

| Supplementary Table 3. Regression of non-exercise model estimating VO_2peak_ in the derivation sample | | | | | |
| --- | --- | --- | --- | --- | --- |
| **Variable** | ***B*** | **t-value** | **p-value** | **Adjusted *R^2^*** | **Standard error of estimate** |
| Constant | 67.584 | 8.64 | < .001 | .59 | 4.03 |
| Age | -0.463 | -4.35 | < .001 |  |  |
| Sex | 6.739 | 6.05 | < .001 |  |  |
| BMI | -0.692 | -4.36 | < .001 |  |  |
| LTPA | 0.075 | 2.50 | .016 |  |  |
| Abbreviations: BMI, body mass index; LTPA, leisure time physical activity; B, unstandardised beta; *R^2^* coefficient of determination from linear regression model; VO_2peak_, volume of oxygen uptake during peak exercise | | | | | |
